# Clinical Audit and Reaudit of Driving Risk Assessment During Leave Risk Discussions Within an Adult Mental Health Inpatient Hospital

**DOI:** 10.1192/bjo.2022.470

**Published:** 2022-06-20

**Authors:** Muredzwa Adelina Muzenda, Zhiyu Loh, Nasr Mahmoud, Aqif Khan, Jatinder Kour, Amechi Nzekwe

**Affiliations:** Tees, Esk and Wear Valley NHS Foundation Trust, Middlesbrough, United Kingdom

## Abstract

**Aims:**

All detained patients should have a leave risk discussion carried out prior to commencement of home leave. Driving risk must be clearly discussed and captured during the assessment. Driving advice as per DVLA guidance must be documented in case notes and discharge summaries. The aim was to audit and reaudit the use and quality of the driving risk assessment during leave risk discussions within adult mental health inpatient wards at one site under the Tees, Esk and Wear Valleys NHS Foundation Trust. The authors hypothesise that compliance to local policies could be improved upon.

**Methods:**

Standards were set based on local policies. The audit was conducted across all adult acute inpatient wards within the identified mental health hospital. All inpatients who went on a period of home leave during their admission and ultimately discharged during the period from 1 April 2021 to 30 April 2021 (initial audit) and 1 October 2021 to 31 October 2021 (reaudit) were assessed. The data were collected using an audit tool. An Excel spreadsheet was used to collate data: specifically driving status of the patient, whether a leave risk discussion which captured driving risk was carried out, and whether DVLA advice was captured on discharge letters.

**Results:**

48 patients (19 were drivers) during the initial audit and 27 patients (9 were drivers) during the reaudit met the inclusion criteria. For the initial audit, overall compliance for leave risk discussion (73%), specifically for driving risk assessment, did not meet target compliance. Only 5% of drivers were given written DVLA guidance on discharge letters. The reaudit showed a 100% compliance in the use and quality of leave risk discussion. 56% of patients had written confirmation of discussion on DVLA driving advice recorded on discharge summary.

**Conclusion:**

There has been significant improvement in the use and quality of leave risk discussion, and documentation of DVLA driving advice on discharge summary during the reaudit.

The results were discussed at the Regional Audit meeting and the Inpatient Leadership Meeting. The following improvement plan was agreed and implemented:

1. Regular communication amongst Multi-Disciplinary Team (MDT) during Leave Risk Discussion. One healthcare professional assigned to inform patient of the advice and capture conversation on case notes.
2. Junior doctor induction to reiterate importance of capturing DVLA advice on discharge letters.
3. MDT to discuss driving risk and advice during discharge meetings. “Driving advice discussion” to be added to discharge meeting checklist.

